# Regulation of *Mycobacterium tuberculosis*-Dependent HIV-1 Transcription Reveals a New Role for NFAT5 in the Toll-Like Receptor Pathway

**DOI:** 10.1371/journal.ppat.1002620

**Published:** 2012-04-05

**Authors:** Shahin Ranjbar, Luke D. Jasenosky, Nancy Chow, Anne E. Goldfeld

**Affiliations:** 1 Immune Disease Institute and Program in Cellular and Molecular Medicine, Children's Hospital Boston, Boston, Massachusetts, United States of America; 2 Department of Pediatrics Harvard Medical School, Boston, Massachusetts, United States of America; 3 Department of Medicine, Harvard Medical School, Boston, Massachusetts, United States of America; University of New Mexico, United States of America

## Abstract

Tuberculosis (TB) disease in HIV co-infected patients contributes to increased mortality by activating innate and adaptive immune signaling cascades that stimulate HIV-1 replication, leading to an increase in viral load. Here, we demonstrate that silencing of the expression of the transcription factor nuclear factor of activated T cells 5 (NFAT5) by RNA interference (RNAi) inhibits *Mycobacterium tuberculosis* (MTb)-stimulated HIV-1 replication in co-infected macrophages. We show that NFAT5 gene and protein expression are strongly induced by MTb, which is a Toll-like receptor (TLR) ligand, and that an intact NFAT5 binding site in the viral promoter of R5-tropic HIV-1 subtype B and subtype C molecular clones is required for efficent induction of HIV-1 replication by MTb. Furthermore, silencing by RNAi of key components of the TLR pathway in human monocytes, including the downstream signaling molecules MyD88, IRAK1, and TRAF6, significantly inhibits MTb-induced NFAT5 gene expression. Thus, the innate immune response to MTb infection induces NFAT5 gene and protein expression, and NFAT5 plays a crucial role in MTb regulation of HIV-1 replication via a direct interaction with the viral promoter. These findings also demonstrate a general role for NFAT5 in TLR- and MTb-mediated control of gene expression.

## Author Summary

The major cause of AIDS deaths globally has been tuberculosis (TB), which is caused by the bacterium *Mycobacterium tuberculosis* (MTb). Co-infection with MTb exacerbates human immunodeficiency virus type1 (HIV-1) replication and disease progression via both innate and adaptive host immune responses to MTb infection. In this report, we present evidence that the transcription factor NFAT5 plays a crucial role in MTb-induced HIV-1 replication in human peripheral blood cells and monocytes. We also show that MTb infection itself stimulates NFAT5 gene expression in human monocytes and that its expression involves the TLR signalling pathway and requires the downstream adaptor proteins MyD88, IRAK1, and TRAF6. This identification of a novel role for NFAT5 in TB/HIV-1 co-infection reveals that NFAT5 is a major mediator of TLR-dependent gene expression and thus provides a potential new therapeutic target for treatment of HIV-1 and possibly other diseases.

## Introduction

*Mycobacterium tuberculosis* (MTb), the causative agent of tuberculosis (TB), is the most common co-infection and cause of death in patients infected with human immunodeficiency virus type 1 (HIV-1) [1], [2]. Direct engagement of pathogen recognition receptors (PRRs) by MTb on mononuclear phagocytes activates signaling cascades that directly induce transcription from the proviral LTR (reviewed in [3]). Furthermore, inflammatory cytokines and chemokines produced by the human host in response to MTb infection activate signal transduction pathways in CD4 T cells and monocytic cells that also result in transcriptional activation of the HIV-1 LTR [4]–[6]. Activation of HIV-1 replication via these MTb-induced pathways ultimately leads to higher viral loads and, in turn, expedited CD4 T cell loss and progression to AIDS ([7], reviewed in [8]–[10]). Furthermore, the progressive immune compromise associated with HIV-1 infection itself is a major cause of latent MTb reactivation, as well as increased susceptibility to primary TB infection ([11]–[15], reviewed in [8]).

The primary PRR on monocytic cells triggered by MTb infection is toll-like receptor (TLR) 2 [16]–[20]. Engagement of TLR2 results in engagement of the adaptor protein MyD88 and the subsequent recruitment of several kinases, including IRAK1 and IRAK4, and the ubiquitin ligase TRAF6 ([21]–[23], reviewed in [10], [24]). TRAF6 activates IκB kinase (IKK) and mitogen-activated protein (MAP) kinases that, in turn, ultimately induce activation of specific transcription factor families, including the NF-κB and AP-1 families, which have been shown to associate with the HIV-1 LTR and to drive its transcription ([22], [25]–[27], reviewed in [10]).

Notably, HIV-1 comprises several subtypes, and the LTR of each subtype is unique with respect to the number and organization of activator binding sites. For example, HIV-1 subtype B, the most highly characterized viral subtype and the primary cause of infection in the Americas, Europe, Japan, and Australia, has two tandem NF-κB motifs in its LTR. By contrast, HIV-1 subtypes C and E, which have spread disproportionately in TB-burdened sub-Saharan Africa and southeast Asia, have three and one NF-κB binding sites, respectively [1], [28]–[30].

We previously showed that the most primordial member of the nuclear factor of activated T cells (NFAT) family, NFAT5 (also known as TonEBP), binds to a site within the HIV-1 LTR that is highly conserved across all HIV-1 subtypes, and is also conserved in HIV-2 and SIV LTRs. This NFAT5 site overlaps the core NF-κB binding motifs in the LTR and is required for constitutive replication of representative HIV-1 subtype B, C, and E isolates in human primary monocyte-derived macrophages (MDM) [31]. Given that NFAT5 has previously been shown to be transcriptionally activated by the MAP kinase p38, which is downstream of MyD88 signaling, [32], we speculated that NFAT5 may also be involved in MTb-induced activation of HIV-1 replication via a TLR-mediated pathway in monocytes and peripheral blood mononuclear cells (PBMC).

Here, we show that NFAT5 and its cognate binding site are of crucial importance for efficient MTb-induced stimulation of HIV-1 replication in human MDM and PBMC. Moreover, we demonstrate that MTb infection increases NFAT5 gene expression in human monocytes in a MyD88-dependent manner. Thus, these results expand the known stimuli of NFAT5 expression to the PRR-mediated innate immune response, and demonstrate that NFAT5 is a critical modulator of MTb-induced enhancement of HIV-1 replication.

## Materials and Methods

### Ethics statement

In our studies we used unidentified human discarded blood cells (peripheral blood mononuclear cells, PBMC), which we obtained from the Blood Bank of Children's Hospital in Boston.

### Cell culture

PBMC from normal unidentified donors were isolated by Ficoll-Hypaque (Pharmacia Corporation, Peapack, NJ) density gradient centrifugation and were cultured in RPMI 1640 medium with 2 mM L-glutamine (BioWhittaker, Inc., Walkersville, MD) supplemented with 10% heat-inactivated fetal calf serum (FCS) (Gemini Bio-Products, www.gembio.com). Human monocytes were isolated from PBMC preparations by positive selection with CD14 microbeads from Miltenyi Biotec (www.miltenyibiotec.com) as described by the manufacturer, and were cultured at 1×10^6^ cells per well in 6-well plates in Macrophage-SFM medium (Gibco, www.invitrogen.com) supplemented with 15 ng/ml recombinant human MCSF (R&D, www.rndsystems.com) and 5% heat-inactivated human AB serum (Nabi, Boca Raton, FL). The cell cultures were incubated at 37°C and 5% CO_2_ for 5 days, after which supernatant was replaced with fresh medium lacking MCSF before manipulation. More than 95% of the adherent cells obtained with this technique were CD14+ macrophages as verified by flow cytometry. THP-1 cells were obtained from ATCC (www.atcc.org) and cultured in RPMI 1640 medium supplemented with 10% FCS (BioWhittaker, www.lonzabio.com). 293T cells were obtained from ATCC (www.atcc.org) and were maintained in Dulbecco's Modified Eagle's medium (DMEM) (Gibco, www.invitrogen.com) supplemented with 10% FCS.

### Viruses

HIV-1_Bal_, HIV-1_Lai_, HIV-1_93TH64_, HIV-1_92TH51_, HIV-1_92TH53_, HIV-1_98CH01_, and HIV-1_98IN22_ were obtained from The Centralized Facility for AIDS Reagents, National Institute for Biological Standard and Control (NIBSC), United Kingdom. HIV-1_KR25_ was isolated in our laboratory as described before [33].

### LTR plasmid construction and reporter assay

LTR reporter plasmids were constructed by inserting nucleotides −208 to +64 relative to the transcriptional initiation site of HIV-1_Lai_, HIV-1_Bal_ (B subtype), HIV-1_98IN17_, HIV-1_98IN22_, HIV-1_98CH01_, HIV-1_CM9_ (C subtype), HIV-1_93TH64_, HIV-1_92TH53_, HIV-1_92TH51_, and HIV-1_KR25_ (E subtype) into the reporter vector pGL3 (Promega BioSciences, www.promega.com) using *Xho I* and *Hind III* restriction enzyme sites. Sequences were aligned and analyzed with CLUSTAL W (www.ebi.ac.uk/clustalw/). The HIV-1_Lai_ NFAT5 binding site-mutant (N5-Mut) reporter plasmid was created by standard PCR-based mutagenesis methods [34]. THP-1 cells (0.8×10^6^/ml) were transfected with 0.3 µg/ml LTR wild-type (WT) or mutated reporter plasmids in combination with 0.03 µg/ml *Renilla* luciferase (pRL-TK) control vector using Effectene transfection reagent (Qiagen; www.qiagen.com). Cells were incubated at 37°C for 16 hours after which they were stimulated with 10 µg/ml MTb CDC1551 lysate or left unstimulated for 8 hours. Reporter gene expression was quantitated by dual-luciferase reporter assay according to the manufacturer's protocol (Promega; www.promega.com).

### Quantitative DNase I footprinting

Recombinant NFAT5 (amino acids 175–471) with an N-terminal 6× His tag was expressed in *E. coli* BL21(DE3) cells (Stratagene; www.stratagene.com) and purified under native conditions using Ni-NTA agarose (Qiagen). Recombinant p50 and p65 were purchased (Active Motif, www.activemotif.com). Quantitative DNase I footprinting was performed as previously described [31].

### HIV-1 infectious molecular clones

The plasmid encoding the full-length infectious molecular clone of HIV-1_Lai_ was obtained from the NIH AIDS Reagent and Reference Program. The HIV-1_Lai/Bal-Env_ infectious molecular clone was constructed by replacing the envelope (env) gp160 amino acids 103–717 of the HIV-1_Lai_ (B subtype that utilizes CXCR4) molecular clone with the corresponding region of HIV-1_Bal_ (B subtype that utilizes CCR5). The HIV-1_Lai/Bal-Env_ chimeric virus uses CCR5 as a secondary receptor. The infectious molecular clone of HIV-1_98IN22_ was constructed using DNA extracted from PBMC that were infected with a primary isolate of HIV-1_98IN22_. HIV-1_Lai/Bal-Env_ and HIV-1_98IN22_ mutant viruses were constructed by introducing point mutations using standard PCR-based mutagenesis methods.

### siRNA transfection of MDM

An siRNA was constructed (Ambion Inc., www.ambion.com) to target a sequence unique to the NFAT5 transcript: 5′-CAACATGCCTGGAATTCAA-3′ (nt 335 to 353) [31]. As described, a control for non-specific siRNA effects, we used an siRNA targeting the green fluorescent protein (GFP), 5′- GGCTACGTCCAGGAGCGCACC-3′. MDM were transfected in 6-well plates using 1 µM of the indicated siRNA in siPORT NeoFX transfection reagent (Ambion Inc., www.ambion.com), prepared as recommended by the manufacturer, in a final volume of 750 µl in Macrophage-SFM medium plus 5% heat-inactivated human AB serum. The cultures were left at 37°C overnight after which cells were washed and incubated in fresh medium. MDM were transfected two times for efficient knock down of NFAT5 expression before infection experiments were performed [31].

### Stable THP-1 cells expressing shRNA

The lentiviral plasmid pLKO.1 expressing shRNA targeting human MyD88 was purchased from Open Biosystems (www.openbiosystems.com) and was validated in our laboratory. shRNA targeting human IRAK1 (forward primer 5′-CCGGAGCAGCTGTCCAGGTTTCGTCTCATAAAACCTGGACAGCTGCTCCTTTTTG-3′, reverse primer 5′-AATTCAAAAAGGAGCAGCTGTCCAGGTTTTATGAGACGAAACCTGGACAGCTGCT-3′ mRNA (IRAK1 mRNA target sequence is underlined) and human TRAF6 (forward primer 5′-CCGGAGAAACCTGTTGTGATTCGTCTCATAAATCACAACAGGTTTCTCCTTTTTG-3′, reverse primer 5′-AATTCAAAAAGGAGAAACCTGTTGTGATTTATGAGACGAATCACAACAGGTTTCT-3′ (TRAF6 mRNA target sequence is underlined) were designed in our laboratory and were cloned into the pLKO.1 plasmid. Lentiviruses encoding shRNA sequences were generated by transfecting the packaging cell line HEK-293T with the shRNA-encoding pLKO.1 plasmids in combination with the packaging plasmid psPAX2 and the envelope plasmid pMD2.G using Effectene transfection reagent (Qiagen, www.qiagen.com). Supernatants were collected 48 hours post-transfection, clarified by centrifugation, and stored at −80°C. THP-1 cells were transduced with the lentiviral particles by culturing the cells with supernatants from the virus-producing cells in the presence of 8 µg/ml polybrene (Millipore, www.millipore.com) and spinoculation for two hours at 2000 RPM. Successfully transduced cells were selected and expanded by treatment with 0.8 µg/ml puromycin.

### MTb culture

The MTb clinical strain CDC1551 was prepared by adding 100 µl of frozen bacteria stock into 10 ml of Middlebrook 7H9 medium (Difco BD, www.bd.com) supplemented with albumin dextrose complex (ADC) and 0.05% Tween 80 (Sigma-Aldrich, www.sigmaaldrich.com). The cultures were grown to an OD_650_ of 0.4 at 37°C to ensure that they were in the logarithmic growth phase. Bacteria were then plated, washed with PBS, resuspended in PBS, and passed through a 5 µm filter to ensure that the bacteria were in a single cell suspension. Bacterial cell numbers were determined by measurement of OD_650_ before further dilution with RPMI 1640 medium for cell infection studies at 10∶1 PBMC∶ bacilli or 1∶1 MDM∶ bacilli and THP-1∶bacilli. Colony-forming unit (CFU) analysis was performed and on days 4 and 7 the average CFU counts were 6×10^3^ and 5×10^4^, respectively, confirming that mycobacteria levels increased over the course of infection of primary MDM.

### Western blot

Whole cell extracts were collected with lysis buffer containing 150 mM NaCl, 50 mM Tris–HCl, pH 7.5, 1% Triton, 10% glycerol, and 1 tablet of Complete EDTA-free Protease Inhibitor Cocktail (Roche) per 25 ml of buffer. Extracts were boiled for 5 min in 1× Laemmli sample buffer with 5% v/v 2-mercaptoethanol and proteins were separated by SDS-PAGE. The gel was transferred to a nitrocellulose Trans-Blot Transfer Membrane (BioRad). The blot was then blocked for 1 h at 37°C in a solution of 4% BSA (Sigma) and 0.1% Tween-20 (BioRad) in a buffer containing 50 mM Tris and 150 mM NaCl at pH 7.6 (BSA/TBST). Primary incubation was carried out with a1∶200 dilution of rabbit anti-NFAT5 antibody (H-300) (Santa Cruz Biotechnology) and a 1∶500 dilution of goat anti-Lamin-B1 antibody (sc-6217; Santa Cruz Biotechnology) in BSA/TBST for 2 h at room temperature. The blot was washed 3×5 min in TBST and incubated in 1∶6000 donkey anti-goat-HRP (Santa Cruz Biotechnology) or goat anti-rabbit-HRP (BioRad) as appropriate for 1 h. The blot was again washed 3×5 min in TBST and developed with SuperSignal West Pico Chemiluminescent Reagent (Pierce).

### Quantitative PCR

The mRNA expression levels were determined by SYBR Green-based real-time PCR (Applied Biosystems, www.appliedbiosystems.com). The reaction conditions were 95°C for 10 min followed by 40 cycles of 95°C for 15 sec and 60°C for 1 min. The results were normalized using β-actin mRNA as an internal control and expressed as relative values.

### Statistical analysis

Where applicable, results are expressed as mean ± SEM. Comparison between two groups was performed using the paired Student t-Test with the aid of Microsoft Excel software. p≤0.05 was considered significant.

## Results

### MTb increases HIV-1 LTR activity of HIV-1 subtypes B, C, and E

**Figure 1:**
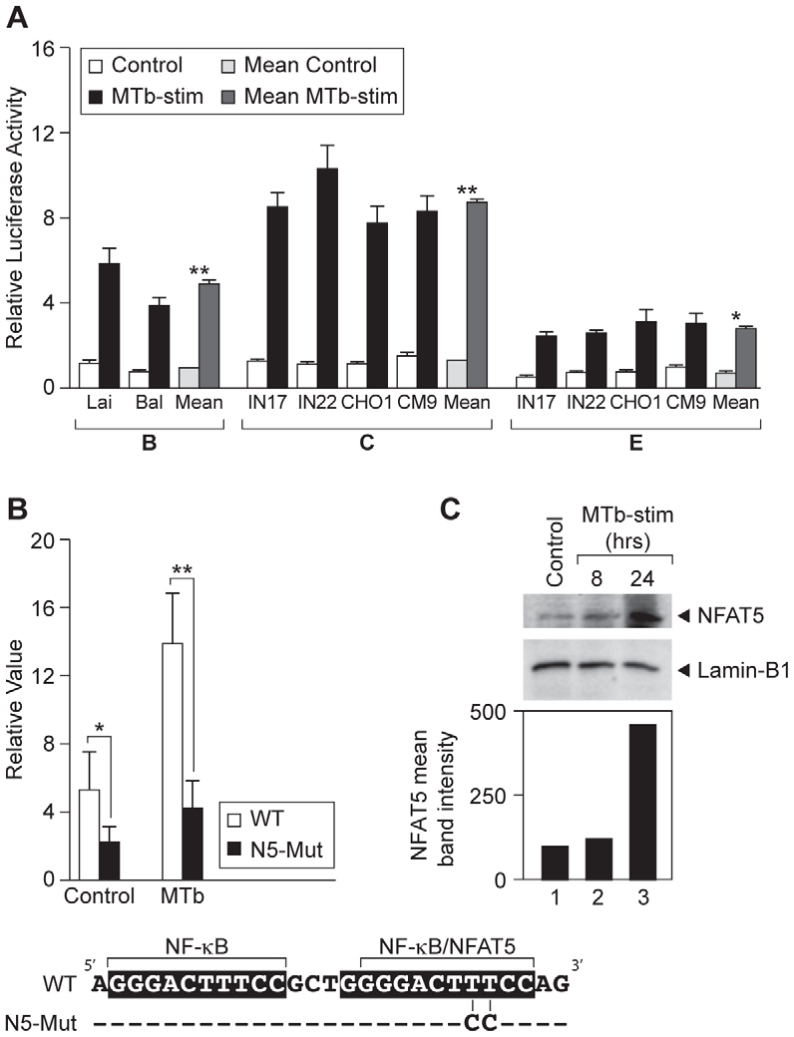
NFAT5 interaction with the LTR is important for MTb-induced HIV-1 transcription. (A) MTb stimulation increases activity of LTRs derived from HIV-1 subtypes B, C, and E. HIV-1 LTRs (−208 to +64 nt relative to the transcription start site) from representative subtype B, C, and E viral isolates were cloned into plasmid pGL3. THP-1 cells (0.8×10^6^/ml) were transfected with each reporter plasmid (0.3 µg/ml) plus the *Renilla* luciferase control plasmid pRL-TK (0.03 µg/ml) and incubated at 37°C for 16 hours. Cells were then either left untreated or treated with 10 µg/ml MTb lysate for 8 hours before termination of the cultures. In the histogram, open bars represent individual LTR activities in untreated cells. Light grey bars represent mean values of LTR activities from each subtype in untreated cells. Black bars represent individual LTR activities in MTb lysate-treated cells, and dark grey bars represent mean values of LTR activities from each subtype in cells treated with MTb lysate. LTR transcriptional activity for all of the representative LTRs tested was significantly increased in cultures treated with MTb lysate in comparison to untreated cultures. Results are from three independent experiments performed in duplicate (*, p<0.05; **, p<0.01 as compared to unstimulated cultures). (B) Specific disruption of the NFAT5 binding site significantly reduces LTR-reporter gene activity in monocytic cells in response to MTb lysate treatment. THP-1 cells were transfected with luciferase expression vectors encoding nucleotides 208 to +64 of the wild-type HIV-1_Lai_ LTR and an HIV-1_Lai_ LTR containing the NFAT5 binding site mutations (N5-Mut). After 16 hours, the cells were left untreated or exposed to 10 µg/ml MTb lysate for 8 hours at 37°C. Disruption of NFAT5 binding to the enhancer region significantly suppressed LTR-driven reporter gene expression in comparison to the wild-type LTR when cells were treated with MTb lysate (p<0.01). LTR activity was also suppressed in the untreated cells but to a lesser extent (p<0.05). Results are from three independent experiments performed in duplicate and adjusted to *Renilla* luciferase control expression (*, p<0.05; **, p<0.01). Nucleotide sequences representing the wild-type and NFAT5 binding site-mutated HIV-1_Lai_ LTRs are shown at the bottom of the figure. (C) MTb lysate increases NFAT5 protein levels in monocytic cells. THP-1 cells were left untreated (control) or exposed to 10 µg/ml MTb lysate for 8 or 24 hours at 37°C. Whole cell extracts were collected and analyzed by western blot with anti-NFAT5 antibody. An antibody directed against Lamin-B1 was used as a loading control. The histogram shows densitometric analysis of the NFAT5 bands from the western blot autoradiograph displayed and values represent mean band intensities at 0, 8, and 24 hours post-stimulation with MTb lysate.

To compare the functional impact of MTb stimulation on subtype-specific HIV-1 LTR activity, we first constructed reporter plasmids containing viral subtype B, C, and E LTRs (−208 to + 64 nt relative to the transcription start site) linked to the firefly luciferase reporter gene. After transfection of the monocytic THP-1 cell line with these plasmids, cells were stimulated with an irradiated whole cell lysate of MTb (H37Rv). We note that MTb lysate induces inflammatory responses in monocytes that resemble those induced in response to live MTb (see for example, [35]–[37]). Upon stimulation, the B, C, and E LTR-driven reporters demonstrated a significant enhancement in luciferase activity (Figure 1A) and the magnitude of this effect was subtype-specific. Subtype C LTRs displayed the strongest activity, while the LTRs from subtype E isolates consistently showed the weakest activity (Figure 1A), consistent with previous studies demonstrating subtype-specific LTR activity that used TNF as a stimulus [38], [39].

### Specific disruption of NFAT5 binding impairs LTR-driven transcription in THP-1 cells in response to MTb stimulation

Although the transcription factor NFAT5 binds to a core NF-κB binding motif in the HIV-1 LTR enhancer region of subtype B, when two thymines (TT) are changed to cytosines (CC) in the proximal NF-κB binding motif (named N5-Mut) (bottom of Figure 1B), the binding of NFAT5 can be disrupted while leaving binding of NF-κB unperturbed [31]. We examined the requirement of NFAT5 for MTb-induced LTR activity by transfecting a wild-type and NFAT5 mutant LTR reporter construct into THP-1 cells, followed by stimulation with an MTb lysate. As shown in Figure 1B, in the absence of MTb lysate stimulation the activity of the NFAT5 binding site-mutant LTR was significantly reduced (p<0.05) in comparison to the wild-type LTR. When the NFAT5 binding site-mutant LTR was examined in THP-1 cells stimulated with the MTb lysate, its activity was reduced to an even more significant extent (p<0.01).

To determine whether MTb lysate stimulation directly enhances NFAT5 expression in THP-1 cells, we stimulated cells for 8 or 24 hours or left them unstimulated and examined NFAT5 protein levels by western blot. As shown in Figure 1C, we found that NFAT5 protein levels steadily increased in response to MTb lysate stimulation, revealing that TLR engagement by MTb results in enhanced levels of NFAT5, consistent with its playing a role in MTb-induced activation of the HIV-1 LTR.

### MTb increases NFAT5 mRNA levels in human MDM

**Figure 2:**
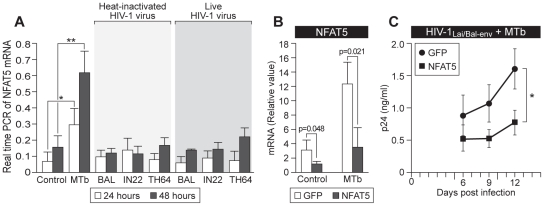
MTb induces NFAT5 gene expression and knockdown of NFAT5 impairs HIV-1 replication during MTb co-infection. (A) MTb but not HIV-1 increases NFAT5 mRNA synthesis in human macrophages. MDM were isolated from PBMC obtained from four normal donors. The cells were incubated for 24 or 48 hours with heat-inactivated or live R5-tropic HIV-1 subtype B, C, or E isolates, live MTb strain CDC1551 (1∶1 MDM∶bacilli), or left untreated. Quantitative real-time PCR analysis of NFAT5 mRNA expression levels revealed that the low, constitutively present level of NFAT5 mRNA expressed in untreated cells was not affected by exposure to live or inactivated HIV-1, but was significantly increased at both 24 (*, p<0.05) and 48 (**, p<0.01) hours following infection with MTb. (B) NFAT5-specific siRNA effectively suppresses NFAT5 expression in MDM in both the absence and presence of MTb co-infection. MDM (1×10^6^ cells/well in a 6-well plate) from four normal donors were transfected with siRNA specific for NFAT5 or, as a control, GFP, and were then infected with MTb (1∶1 MDM∶bacilli) or left uninfected. In the cells transfected with control siRNA, MTb infection significantly increased NFAT5 expression in comparison to uninfected cells (open bars). When MDM were transfected with NFAT5-specific siRNA, both constitutively expressed (p = 0.048) and MTb-induced (p = 0.021) NFAT5 mRNA were significantly suppressed (grey bars). (C) HIV-1 subtype B replication is significantly suppressed in the presence of MTb co-infection when NFAT5 expression is abrogated in human MDM. MDM from four normal donors were transfected with NFAT5-specific and control siRNAs as described in 2B. Cells were then infected with HIV-1_Lai/Bal-env_ followed by co-infection with MTb CDC1551. Virus replication was measured at days 6, 9, and 12 post-infection. At day 12, virus replication was significantly reduced in cells transfected with NFAT5-specific siRNA in comparison to cells transfected with control siRNA (*, p<0.05). We monitored the morphology of cells within the infected cultures and did not observe noticeable macrophage necrosis even at the final timepoint of viral harvest.

Given that macrophages, which are the primary target of MTb infection, are also a major reservoir of HIV-1 as infection progresses [40], we next investigated whether MTb is able to directly enhance NFAT5 mRNA expression in primary human MDM. We prepared MDM from five normal donors, stimulated the cells with the MTb lysate or left them unstimulated, and measured NFAT5 gene expression levels by quantitative real-time PCR at 24 and 48 hours. We also investigated whether HIV-1 infection is capable of inducing NFAT5 mRNA synthesis by infecting MDM with live or heat-inactivated R5-tropic representatives of subtype B (HIV-1_Bal_), C (HIV-1_98IN22_), or E (HIV-1_92TH64_). As shown in Figure 2A, stimulation with MTb lysate significantly increased NFAT5 mRNA levels at 24 (p<0.05) and 48 (p<0.01) hours, whereas infection with viable or heat-inactivated HIV-1 isolates did not increase NFAT5 mRNA levels (Figure 2A). Thus, MTb specifically enhances NFAT5 mRNA expression in human MDM, and this response continues to increase for at least 48 hours post-stimulation (Figure 2A).

### RNAi directed against NFAT5 inhibits replication of subtype B HIV-1

To extend the results we obtained in the reporter assays to a physiological TB/HIV co-infection model, we next tested the effect of siRNA-mediated ablation of NFAT5 mRNA levels in MDM co-infected with a subtype B HIV-1_Lai_ infectious molecular clone and a clinical isolate of MTb. To perform this experiment, we first constructed an HIV-1_Lai_ clone bearing the CCR5-tropic envelope region of HIV-1_Bal_ (HIV-1_Lai/Bal-env_) so that it would efficiently infect primary MDM. We used this approach to ensure that our analysis of the roles of NF-κB and NFAT5 in MTb-induced HIV-1 replication could be interpreted in the proper context of previous research findings that examined LTR regulation in the context of full-length viral replication [41]–[46]. The HIV-1_Lai/Bal-Env_ infectious clone is isogenic for the entire sequence of the parental HIV-1_Lai_ infectious clone except for the substitution of the HIV-1_Bal_ envelope co-receptor binding region in place of the HIV-1_Lai_ envelope co-receptor binding region. We note that we confirmed that HIV-1_Lai/Bal-Env_ grew in PBMC at a similar rate to wild-type HIV-1_Bal_, indicating proper co-receptor engagement and internalization of this infectious clone (data not shown).

Next, we knocked down NFAT5 mRNA levels in MDM using an siRNA that suppresses both NFAT5 mRNA and NFAT5 protein levels [31]. As shown in Figure 2, transfection of the siRNA specific for NFAT5 reduces NFAT5 mRNA levels in both MTb-uninfected (p = 0.048) and MTb-infected (p = 0.021) MDM as compared to transfection of control GFP siRNA into MTb-uninfected or -infected MDM (Figure 2B). We note that although siRNA normally is effective for 48–72 hours in cell lines that divide rapidly, in human MDM, which are non-dividing cells, siRNA to host factors remains detectable and functional up to at least 15 days post-transfection [47].

MDM in which NFAT5 expression had been inhibited with NFAT5 siRNA or that were transfected with control GFP siRNA were then infected with 1000 TCID_50_ of HIV-1_Lai/Bal-env_. After overnight virus infection, the cells were then co-infected with the MTb clinical strain CDC1551. Free virus levels were then measured in culture supernatants from MDM transfected with NFAT5-specific siRNA or GFP control siRNA at days 6, 9 and 12 post-HIV-1 infection to measure the impact of NFAT5 inhibition on MTb-induced HIV replication.

As shown in Figure 2C, HIV-1 replication was suppressed at days 6 and 9 post-infection in the NFAT5 siRNA-treated cells as compared to cells treated with control siRNA, and it was significantly inhibited by day 12 post-infection (p<0.05) (Figure 2C). Thus, knock down of NFAT5 expression significantly impairs HIV-1 subtype B replication in MDM co-infected with MTb.

### NF-κB and NFAT5 binding to the HIV-1 LTR can be specifically disrupted

To demonstrate that the impact of NFAT5 silencing on MTb-induced viral replication was a direct effect due to modulation of recruitment of NFAT5 to the HIV-1 LTR and not due to secondary, NFAT5-regulated effects, we set out to disrupt NFAT5 binding to the viral LTR in the context of HIV-1/MTb co-infection. This series of experiments also allowed us to dissect the relative importance of NFAT5 and NF-κB binding to the HIV-1 LTR in MTb regulation of HIV-1 replication.

**Figure 3:**
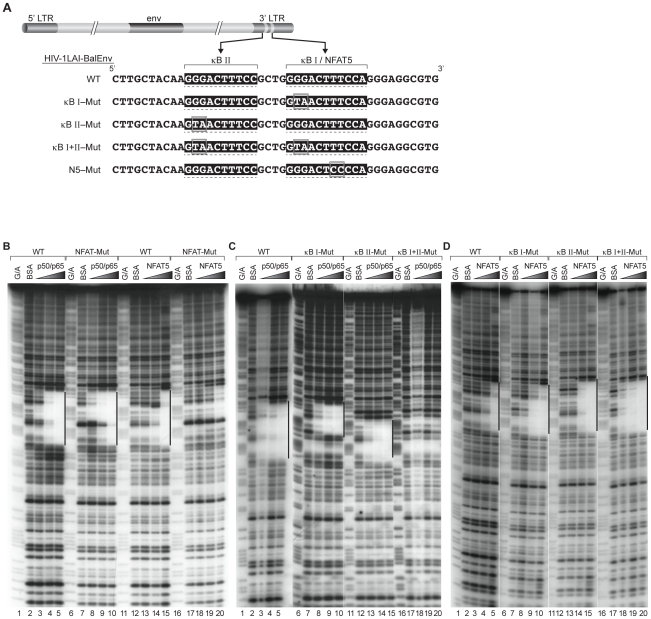
Specific disruption of NFAT5 or NF-κB binding sites in the LTR of HIV-1 subtype B. (A) Mutations introduced into a HIV-1_Lai/Bal-env_ infectious molecular clone. NF-κB and NFAT5 binding site mutations were introduced into the LTR of the HIV-1_Lai/Bal-env_-wild type (WT) infectious molecular clone. The NF-κB binding site mutants (HIV-1_Lai/Bal-env_-κB I-Mut, HIV-1_Lai/Bal-env_-κB II-Mut, HIV-1_Lai/Bal-env_-κB I+II-Mut), and NFAT5 binding site mutant (HIV-1_Lai/Bal-env_-N5-Mut) are shown along with the HIV-1_Lai/Bal-env_-WT sequence. Mutations were introduced into the 3′ LTR of the HIV-1_Lai/Bal-env_–WT proviral sequence. (B) Specific mutation of the NFAT5 binding site abolishes NFAT5 binding to the viral LTR but does not affect NF-κB p50/p65 binding to the overlapping NF-κB binding site. Quantitative DNaseI footprinting analysis is shown using HIV-1 LTR fragments (-262 to +4 nt relative to the transcription start site) from HIV-1_Lai/Bal-env_-WT and HIV-1_Lai/Bal-env_-N5-Mut and increasing concentrations of recombinant NF-κB (p50/p65) (25 ng, 100 ng, and 500 ng), or NFAT5 (10 ng, 50 ng, and 250 ng). The regions that are protected from DNase I cleavage by the binding of NF-κB and NFAT5 are indicated with a bars. (C) Specific disruption of the HIV-1 subtype B NF-κB binding sites effectively abrogates recombinant p50/p65 binding. Quantitative DNaseI footprinting analysis is shown of nucleotides −262 to +4 from HIV-1_Lai/Bal-env_-WT, HIV-1_Lai/Bal-env_-κB I-Mut, HIV-1_Lai/Bal-env_-κB II-Mut, and HIV-1_Lai/Bal-env_-κB I+II-Mut and increasing concentrations of recombinant NF-κB (p50/p65) (25 ng, 100 ng, and 500 ng). The regions that are protected from DNase I cleavage by the binding of recombinant NF-κB are indicated with a bars. (D) Specific disruption of the HIV-1 subtype B NF-κB binding site does not inhibit but enhances NFAT5 binding to this region. Quantitative DNaseI footprinting analysis is shown of nucleotides −262 to +4 from HIV-1_Lai/Bal-env_-WT, HIV-1_Lai/Bal-env_-κB I-Mut, HIV-1_Lai/Bal-env_-κB II-Mut, and HIV-1_Lai/Bal-env_-κB I+II-Mut and increasing concentrations of recombinant NFAT5 (10 ng, 50 ng, and 250 ng). The regions that are protected from DNase I cleavage by the binding of recombinant NFAT5 are indicated with a bars.

To perform these experiments, we constructed a panel of infectious HIV-1_Lai/Bal-env_ molecular clones where either the NFAT5 site was specifically disrupted or the two NF-κB binding sites were either individually or dually disrupted (Figure 3A). Specifically, we constructed a clone in which the NFAT5 binding site was mutated (named HIV-1_Lai/Bal-env_-N5-Mut) by changing the CC dinucleotide to TT in the core NFAT5 binding site and introduced substitution mutations into the NF-κB/NFAT5 shared binding element that we predicted would disrupt NF-κB binding but preserve NFAT5 binding. Specifically, we mutated two guanines (GG) in the κB I site and changed them to thymine and adenine (TA) (HIV-1_Lai/Bal-env_-κB I-mut) and also introduced the same GG to TA change in the second, more distal NF-κB site (κB II) in HIV-1_Lai/Bal-env_ (HIV-1_Lai/Bal-env_-κB II-Mut). We also created a double NF-κB site mutant virus (HIV-1_Lai/Bal-env_-κB I+II-Mut) in order to test the impact of complete disruption of NF-κB binding to the HIV-1 LTR on MTb regulation.

To determine the specificity of these substitutions upon NFAT5 and NF-κB p50/p65 binding to the LTR, we performed a quantitative DNase I footprinting analysis using the wild-type LTR or mutant LTRs in combination with increasing concentrations of recombinant NFAT5 or NF-κB p50/p65 proteins. As shown in Figure 3B, the N5-Mut LTR could not bind NFAT5 (compare lanes 12–15 and lanes 17–20), but p50/p65 binding was not impaired (compare lanes 2–5 with lanes 7–10). By contrast, changing the GG dinucleotide to TA in either NF-κB binding motif, as predicted, inhibited p50/p65 binding at each site (compare lanes 2–5 with lanes 7–10 and lanes 12–15 of Figure 3C). As expected, mutation of both NF-κB sites (κB I+II-Mut) resulted in abrogation of p50/p65 binding to the LTR. However, recombinant NFAT5 binding to the single and the double NF-κB mutant LTRs was not impaired and indeed appeared enhanced (compare lanes 2–5 to lanes 7–20 of Figure 3D). Thus, binding of NFAT5 or NF-κB p50/p65 to their respective motifs can be specifically disrupted within an overlapping or shared binding site.

### NF-κB and NFAT5 binding play independent activating roles in MTb-mediated induction of HIV-1 replication

We next examined the functional impact of specifically disrupting NF-κB or NFAT5 binding on regulation of HIV-1 replication by infecting bulk PBMC from four normal donors with the wild-type or mutant HIV-1_Lai/Bal-env_ molecular clones. After overnight infection, cells were co-infected with MTb (CDC1551) or mock-infected and free virus levels were detected by measuring p24 levels in culture supernatants at days 4, 7, and 12 post-HIV-1 infection.

**Figure 4:**
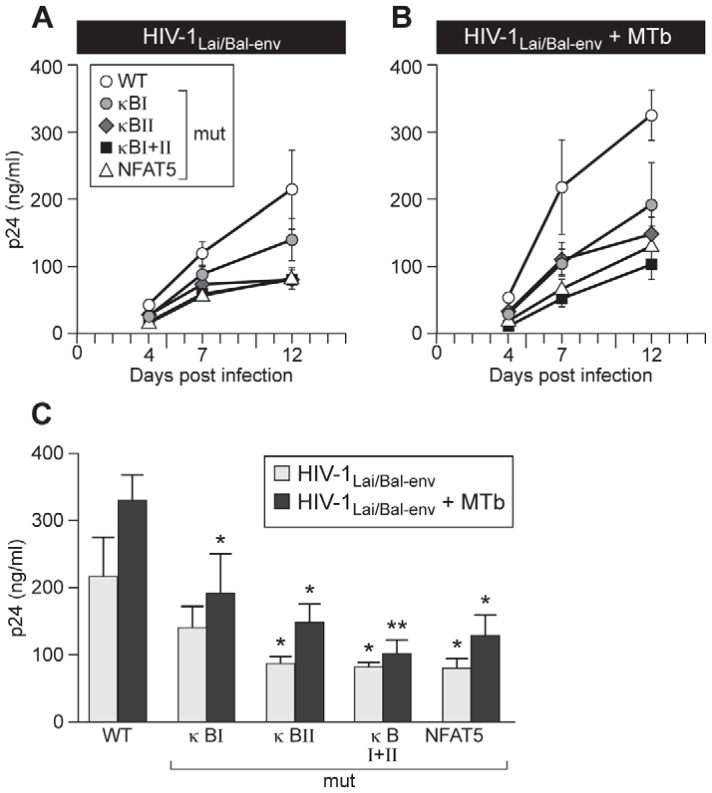
MTb-induced HIV-1 replication in PBMC depends on binding of NFAT5 and NF-κB to the LTR. Specific disruption of the NF-κB or NFAT5 binding sites in the HIV-1 subtype B LTR inhibits R5-tropic virus replication in human PBMC after co-infection with MTb. PBMC from four normal donors were infected with 1000 TCID_50_ HIV-1_Lai/Bal-env_-WT or the mutants HIV-1_Lai/Bal-env_-κB I-Mut, HIV-1_Lai/Bal-env_-κB II-Mut, HIV-1_Lai/Bal-env_-κB I+II-Mut, or HIV-1_Lai/Bal-env_-N5-Mut and either; (A) left untreated, or (B) co-infected with MTb isolate CDC1551 (10∶1 cells∶bacilli). Viral p24 levels in culture supernatants were measured at days 4, 7 and 12 post-infection. (C) Histograms show viral p24 levels at day 12 in the MTb uninfected (grey bars) and MTb co-infected (black bars) PBMC cultures. Replication of the mutant viruses was compared to wild-type virus replication under the same experimental conditions (without and with MTb co-infection, respectively). *, p<0.05; **, p<0.01, as compared to HIV-1_Lai/Bal-Env_-WT.

Replication of each mutant virus was reduced in comparison to wild-type virus in mock- and MTb-co-infected cells at all timepoints examined (Figures 4A–4B). As depicted in the histograms displayed in Figure 4C, at day 12 post-HIV-1 infection in MTb-co-infected cells there was a significant reduction in levels of HIV-1_Lai/Bal-env_-κB I-Mut, HIV-1_Lai/Bal-env_-κB II-Mut, HIV-1_Lai/Bal-env_-κB I+II-Mut, and HIV-1_Lai/Bal-env_ -N5-Mut in comparison to wild-type virus (Figure 4C). In the absence of MTb co-infection, p24 levels were also significantly lower in the PBMC cultures infected with HIV-1_Lai/Bal-env_–κB II-Mut, HIV-1_Lai/Bal-env_-κB I+II-Mut and HIV-1_Lai/Bal-env_-N5-Mut, but not HIV-1_Lai/Bal-env_-κB I-Mut, in comparison to wild-type virus (Figure 4C). None of the mutations completely abolished viral induction by MTb co-infection. However, the replication of each mutant virus was impaired to a more significant extent in the context of MTb co-infection, consistent with important roles for both NF-κB and NFAT5 in MTb-induced HIV-1 replication.

**Figure 5:**
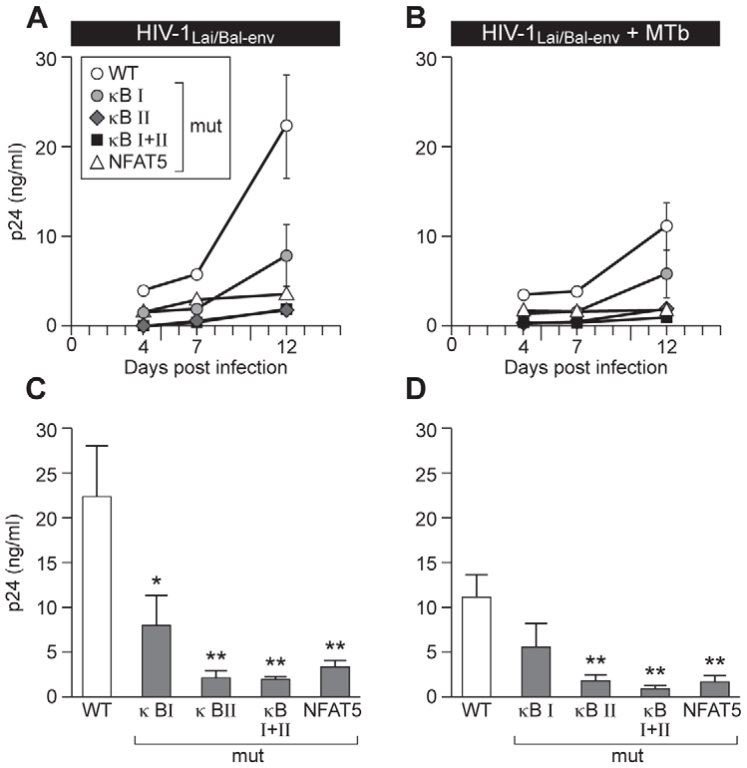
Disruption of NF-κB or NFAT5 sites in the LTR inhibits MTb-induced HIV-1 replication in MDM. MDM from four normal donors were infected with 1000 TCID_50_ of HIV-1_Lai/Bal-env_–WT or the mutants HIV-1_Lai/Bal-env_-κB I-Mut, HIV-1_Lai/Bal-env_-κB II-Mut, HIV-1_Lai/Bal-env_-κB I+II-Mut, or HIV-1_Lai/Bal-env_-N5-Mut, and were either (A) left infected with virus alone or (B) co-infected with MTb CDC1551 (1∶1 MDM∶bacilli). Viral p24 levels in the culture supernatants were measured at days 4, 7 and 12 post-infection. Presentation of viral p24 levels at day 12 in cultures infected with (C) HIV-1 alone or (D) co-infected with MTb are shown as histograms. (*, p<0.05; **, p<0.01, as compared to HIV-1_Lai/Bal-env_–WT).

To specifically examine the functional impact of NF-κB and NFAT5 binding site usage in MDM, which are efficiently infected by both pathogens, we next isolated MDM from four normal donors and infected these cells with 1000 TCID_50_ of wild-type HIV-1_Lai/Bal-env_ or with the mutant viral clones HIV-1_Lai/Bal-env_-κB I-Mut, HIV-1_Lai/Bal-env_-κB II-Mut, HIV-1_Lai/Bal-env_-κB I+II-Mut, or HIV-1_Lai/Bal-env_-N5-Mut. After overnight incubation, cell cultures were co-infected with MTb (CDC1551) or left infected with virus alone. Supernatants were collected at days 4, 7, and 12 post-HIV-1 infection and virus replication was measured. As shown in Figures 5A and 5C, in the absence of MTb co-infection, replication of mutant viral molecular clones (HIV-1_Lai/Bal-env_-κB I-Mut (p<0.05), HIV-1_Lai/Bal-env_-κB II-Mut (p<0.01), and HIV-1_Lai/Bal-env_-κB I+II-Mut (p<0.01) as well as HIV-1_Lai/Bal-env_-N5-Mut (p<0.01) were significantly reduced in comparison to the wild-type virus (Figures 5A–5C).

When the cultures were co-infected with MTb, p24 levels increased when the cells were infected with either the wild-type or, to lower levels, with the κB-I mutant virus (Figure 5B). However, p24 levels were significantly inhibited in cells infected with HIV-1_Lai/Bal-env_ -N5-Mut (p<0.01) (Figure 5D). Moreover, p24 levels were significantly lower at day 12 in cultures infected with HIV-1_Lai/Bal-env_-κB II-Mut and HIV-1_Lai/Bal-env_-κB I+II-Mut as compared to infection of cells with wild-type virus (Figure 5D). Notably, although replication of the mutant virus with the single, proximal NF-κB binding site mutation in the shared NFAT5/NF-κB element (HIV-1_Lai/Bal-env_-κB I-Mut) was diminished, this was not significant at day 12 (Figures 5B–5D).

We note that overall p24 levels were noticeably lower in the cultures co-infected with MTb compared with those infected with HIV-1 alone. This is consistent with previous observations showing that MTb infection of human primary macrophage cultures ex vivo suppresses HIV-1 infection due to chemokine synthesis and the enhanced expression of cellular inhibitory factors [48]–[50]. Given that NF-κB is efficiently activated in primary MDM in response to TLR agonists [51] and as we have shown in Figure 2, NFAT5 gene expression is also induced by TLR agonists, MDM are a suitable experimental system to analyze the effect of NF-κB versus NFAT5 binding site mutations on virus replication in isolated MDM in the absence or presence of MTb co-infection. Taken together, the results from PBMC and MDM co-infection experiments demonstrate that the conserved NFAT5 binding site plays as important a transcriptional role in LTR regulation by MTb as do the NF-κB sites.

### NFAT5 is important for enhancement of HIV-1 subtype C replication in response to MTb infection

**Figure 6:**
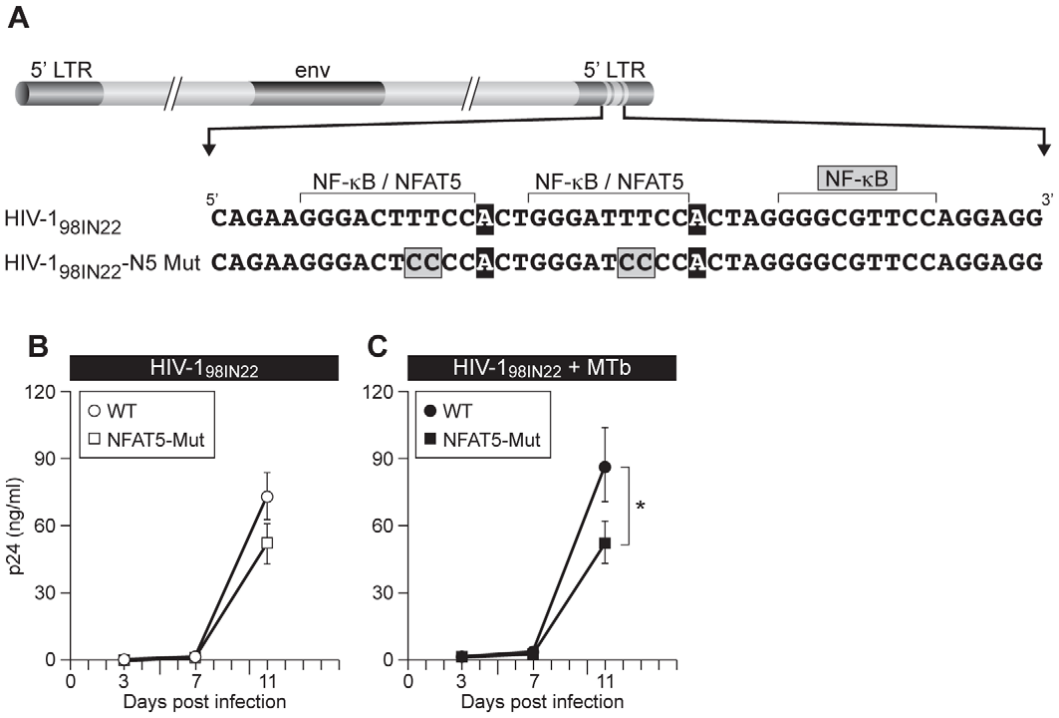
Disruption of the NFAT5 site in HIV-1 subtype C significantly impairs MTb-induced viral replication. (A) Mutation of the NFAT5 binding site in an HIV-1 subtype C infectious molecular clone. NFAT5 binding site mutations were introduced into the LTR of subtype C HIV-1_98IN22_-WT. The NFAT5 binding site mutant (HIV-1_98IN22_ N5-Mut) LTR sequence is shown alongside that of HIV-1_98IN22_-WT. The HIV-1_98IN22_ isolate analyzed here contains three NF-κB and two NFAT5 binding sites. The unique 3′ terminal adenine, which is important for NFAT5 binding to its site, is shown in blocks. Mutations were introduced into the 3′ LTR of the HIV-1_98IN22_-WT proviral sequence. PBMC from four normal donors were infected with 1000 TCID_50_ of HIV-1_98IN22_–WT or HIV-1_98IN22_-N5-Mut. Cells were then (B) left infected with virus alone or (C) co-infected with MTb strain CDC1551 (10∶1 cells∶bacilli). Culture supernatants were collected at days 3, 7 and 11 post-infection and viral p24 levels were measured. Replication of HIV-1_98IN22_-N5-Mut was significantly reduced at day 11 post-infection in comparison to HIV-1_98IN22_–WT (*, p<0.05) in the presence of MTb co-infection.

As shown in Figure 1, the subtype C LTR was the most active of the HIV-1 LTR subtypes in the reporter assays. Subtype C LTRs generally have three functional NF-κB sites in their LTRs, and subtype C is the predominant viral subtype in the African and Asian HIV-1 epidemics where MTb co-infection is extremely common [1], [2], [28], [52]. We thus next extended our analysis of the role of NFAT5 in MTb/HIV-1 co-infection to a subtype C isolate. We first constructed an infectious molecular clone of the HIV-1 subtype C primary isolate HIV-1_98IN22_, which has two NFAT5 binding sites in its LTR and three NF-κB sites (Figure 6A). We disrupted the two NFAT5 binding sites by changing the TT of each site to CC to create a mutant virus that we named HIV-1_98IN22_-N5-Mut. Bulk PBMC from four normal donors were infected overnight with 1000 TCID_50_ of wild-type HIV-1_98IN22_ or HIV-1_98IN22_-N5-Mut and the cultures were then co-infected with MTb CDC1551 or left infected with virus alone. At day 11 post-viral infection, wild-type HIV-1_98IN22_ replication was greater, but not significantly so, as compared to replication of HIV-1_98IN22_ NFAT5-Mut (Figure 6B). However, in the context of MTb co-infection, wild-type HIV-1_98IN22_ replication was significantly increased (p<0.05) at day 11 over HIV-1_98IN22_ NFAT5-Mut replication (Figure 6C), indicating that the absence of NFAT5 binding sites was particularly detrimental to virus replication in the presence of MTb co-infection. Thus, even when three functional NF-κB binding sites are present in the LTR, as in the HIV-1 subtype C infectious clone studied here, disruption of NFAT5 binding to the LTR impairs virus replication in response to MTb co-infection.

### MTb induces NFAT5 gene expression via the MyD88-dependent signaling cascade

**Figure 7:**
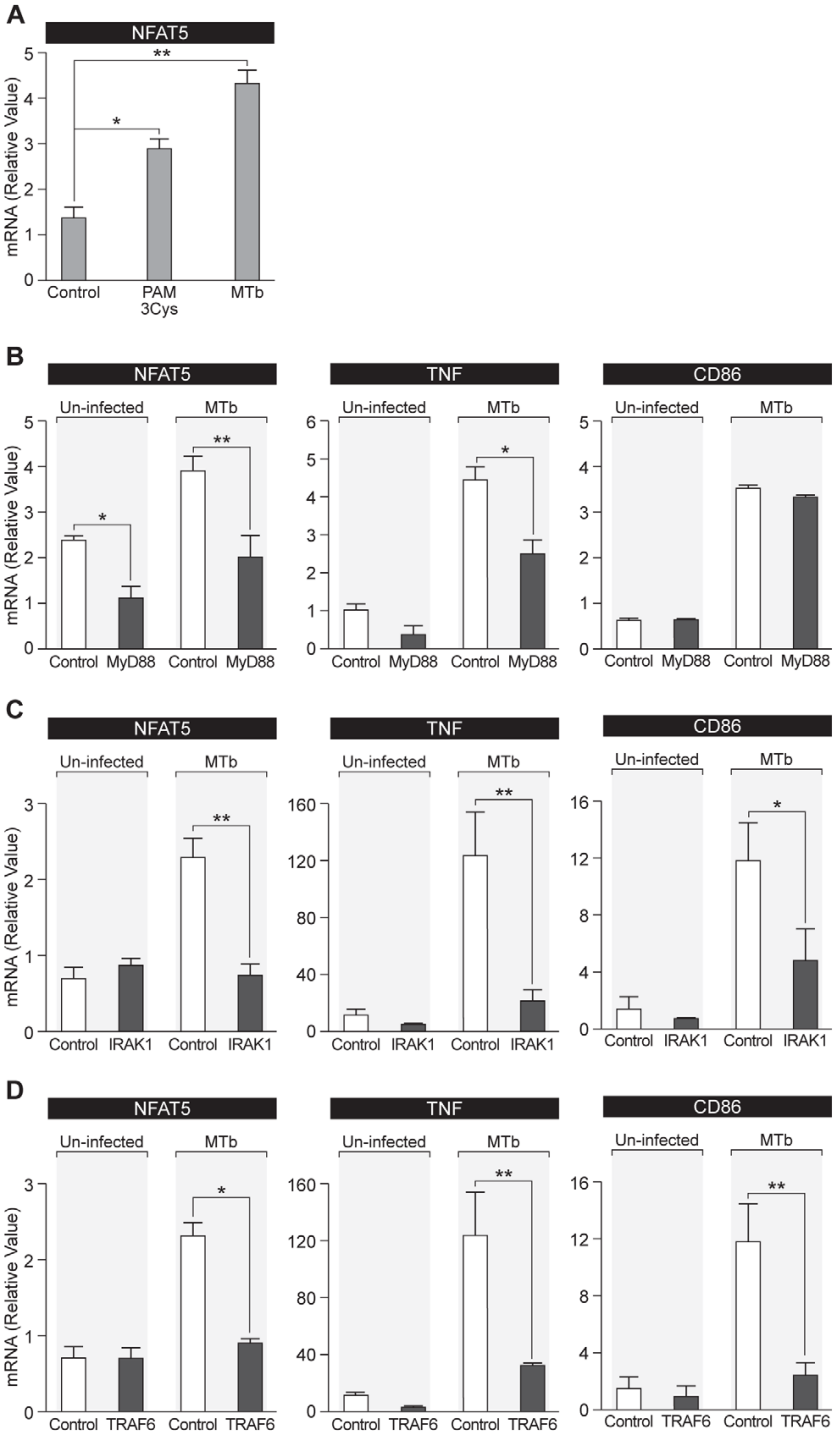
The MyD88-mediated signaling cascade is important for MTb induction of NFAT5 expression in human monocytes. (A) Engagement of TLR2 significantly enhances NFAT5 mRNA synthesis in monocytes. THP-1 cells were incubated with the TLR2-specific ligand Pam3Cys (5 µg/ml), infected with MTb strain CDC1551 (1∶1 cells∶bacilli), or left untreated. After 16 hours, NFAT5 mRNA levels were measured. NFAT5 mRNA expression was significantly increased when the cells were incubated with Pam3Cys (*, p<0.05) or infected with MTb (**, p<0.01). (B) MyD88 is important for MTb induction of NFAT5 gene expression in human monocytes. THP-1 cells that constitutively express lentivirally-delivered shRNA targeting MyD88 or control shRNA targeting GFP were constructed as described in Materials and Methods. Cells were infected with MTb strain CDC1551 (1∶1 cells∶bacilli) or left uninfected for 16 hours at 37°C, and RNA was analyzed. NFAT5 mRNA levels were significantly inhibited in the cells transduced with MyD88-specific shRNA (black bars) compared with the cells transduced with control shRNA (open bars) in both, the absence or presence of MTb infection. TNF and CD86 mRNAs, the expression of which are MyD88-dependent and -independent, respectively, during MTb infection, were measured as controls. (C, D) IRAK1 and TRAF6 are required for MTb induction of NFAT5 gene expression in human monocytes. THP-1 cells that constitutively express lentivirally-delivered IRAK1- or TRAF6-specific shRNA were constructed as described in Materials and Methods. THP-1 cells expressing control shRNA were used as described in 7B. Cells were infected with MTb strain CDC1551 or left uninfected for 16 hours at 37°C, and RNA was analyzed. NFAT5 expression was significantly inhibited in the MTb-infected cells expressing either IRAK1 (C) or TRAF6 (D) shRNA (black bars) compared to cells expressing control shRNA (open bars). TNF and CD86 mRNAs, both of which require IRAK1 and TRAF6 to be induced by PRR activation, were measured as controls. (*, p<0.05; **, p<0.01).

To begin to decipher the cellular mechanisms underlying NFAT5-dependent, MTb-induced HIV-1 replication, we next investigated the requirement for specific signaling molecules in the TLR pathway that are required for MTb stimulation of NFAT5 gene expression. We began by examining the requirement of TLR2 ligation in enhancing NFAT5 mRNA levels. When THP-1 cells were treated with the TLR2-specific agonist Pam3Cys for 16 hours, NFAT5 mRNA levels were significantly increased in Pam3Cys-stimulated cells as compared to mock-stimulated control cells (Figure 7A). As a positive control for this experiment we also infected THP-1 cells with CDC1551 (Figure 7A). Notably, MTb infection enhanced NFAT5 mRNA levels to a greater extent than Pam3Cys stimulation, suggesting that additional PRRs may be triggered during actual MTb infection, augmenting its stimulatory effect on NFAT5 gene expression (Figure 7A).

The adaptor molecule MyD88 transmits signals upon TLR2 ligation (reviewed in [53]). To confirm that this protein and, therefore, that this TLR-dependent pathway is important for MTb induction of NFAT5 gene expression, we constructed a THP-1 cell line that constitutively expresses a lentivirally-delivered short hairpin (sh)RNA targeting MyD88. Real-time PCR analysis demonstrated that MyD88 mRNA levels in these cells were constitutively and significantly reduced in comparison to control THP-1 cells transduced with a lentivirus expressing control GFP shRNA (data not shown). These MyD88 knockdown THP-1 cells were then infected with MTb (CDC1551) and NFAT5 mRNA levels were examined. Since TNF is a MyD88-dependent gene [21], [54], [55], as a positive control we measured TNF mRNA levels. For a negative control, we measured mRNA levels of the MyD88-independent gene CD86 [55], [56]. As shown in Figure 7B, knockdown of MyD88 specifically inhibited MTb-induced NFAT5 and TNF mRNA levels and had no impact on CD86 mRNA levels. Thus, MTb regulation of NFAT5 is dependent upon MyD88.

To futher dissect the pathway mediating MTb upregulation of NFAT5 transcription, we also constructed THP-1 cells transduced with lentivirally-delivered shRNAs targeting IRAK1 or TRAF6, two signaling molecules downstream of MyD88. As shown in Figures 7C and 7D, knockdown of both IRAK1 and TRAF6 significantly inhibited MTb induced NFAT5 mRNA levels. As expected, MTb-induced TNF gene expression was also significantly reduced (Figures 7C–7D), consistent with a role for IRAK1 and TRAF6 in TNF gene expression secondary to MTb infection as previously reported. Although CD86 gene expression did not depend on MyD88 (Figure 7B), abrogation of IRAK1 and TRAF6 gene expression significantly reduced CD86 mRNA levels, indicating that CD86 expression is IRAK1- and TRAF6-dependent, but MyD88-independent in MTb-infected monocytes (Figures 7C–7D). Taken together, these results clearly demonstrate that MTb upregulation of NFAT5 gene expression in human monocytic cells requires activation of MyD88 and the MyD88-associated adaptor molecules IRAK1 and TRAF6.

## Discussion

The transcription factor NFAT5 is the most evolutionarily divergent member of the Rel family of NFAT proteins [57]. Unlike the other NFAT proteins (NFATp, NFATc, NFAT3, and NFAT4), NFAT5 binds DNA as an obligate dimer in a fashion resembling NF-κB proteins [58], is calcineurin independent, and does not cooperate with the basic region-leucine zipper proteins Fos and Jun in gene activation ([59], [60], reviewed in [61]–[63]). To date, osmotic stress, integrin activation, and T cell-stimulation have been shown to regulate NFAT5 activity [59], [64], [65]. Based on our demonstration here that the host innate immune response to MTb infection induces MyD88-dependent upregulation of NFAT5 gene expression, we have expanded this list to include MTb as an additional stimulus. We have linked this observation to MTb-stimulated HIV-1 gene expression by showing that MTb enhances the replication of HIV-1 subtypes B and C via a direct interaction between NFAT5 and the viral promoter. Thus, HIV-1 has co-opted NFAT5, which is induced as part of the innate immune response to TB, for enhanced viral transcription/replication.

NF-κB is activated after MTb engagement of PRRs [20], [66]. Multiple studies of HIV-1 activation have shown that initial recruitment of the NF-κB p50/p65 heterodimer to the HIV-1 proviral enhancer region is crucial for HIV gene transcription (reviewed in [67], [68]). Upon interaction with the viral LTR, NF-κB rapidly induces hyperacetylation of histones associated with nucleosome 1 (nuc-1) at the HIV-1 transcription start site, resulting in recruitment of the pTEFb complex, which is required for RNA pol II processivity ([69], reviewed in [68], [70]).

In this report we directly examined the relative roles of NFAT5 and NF-κB p50/p65 in HIV-1 replication under conditions of activation by MTb co-infection, when NF-κB levels in the nucleus are elevated. We found that specific disruption of the NFAT5 binding site(s) in R5-tropic subtype B or subtype C infectious molecular clones significantly reduced virus replication in PBMC or MDM co-infected with a clinical isolate of MTb. Thus, NF-κB binding to the viral LTR is not sufficient to compensate for the loss of NFAT5 binding to the LTR under conditions of MTb co-infection. Reciprocally, an intact NFAT5 site could not compensate for disruption of the two NF-κB sites. Thus, both factors are required for MTb-induced HIV-1 replication.

Intriguingly, both in the absence and presence of MTb co-infection, disruption of NF-κB site II consistently resulted in greater suppression of virus replication than disruption of NF-κB site I. Consistent with these findings, synthetic reporter assays have previously shown that these two sites have distinct roles in driving transcription. The downstream NF-κB site I binding site, which is directly upstream of three highly conserved Sp binding sites, appears to enhance Sp protein recruitment to the LTR [71]–[73]. Because NF-κB site I overlaps the NFAT5 binding site, it is possible that loss of NF-κB binding to this site is mitigated, at least in part, by increased NFAT5 binding to the mutated site. This hypothesis is consistent with our quantitative DNase I footprinting analysis that shows that specific disruption of NF-κB site I results in enhanced binding of NFAT5 to the LTR.

HIV-1 subtype C now makes up greater than 50% of all HIV-1 infections worldwide, and its prevalence is especially high in regions of endemic TB [1], [2], [28], [52], Most HIV-1 subtype C isolates possess three NF-κB binding sites in the LTR [30]. Our analysis of MTb whole lysate-stimulated, LTR-driven reporter gene expression from representative B, C, and E LTRs showed that LTRs derived from subtype C isolates were superior in driving reporter gene expression as compared to LTRs derived from subtype B or E isolates. Other studies have found that, in response to TNF, subtype C LTRs drive reporter gene transcription more strongly than other subtype LTRs [38], [39]. In the co-infection model utilized in this report, in which clinical isolates of both MTb and HIV-1 subtype C were used to infect freshly prepared human peripheral blood cells, we found that specific disruption of NFAT5 binding to the LTR significantly impaired viral replication during MTb co-infection, indicating that even the presence of three intact NF-κB binding sites, which is typical for HIV-1 subtype C isolates [30], cannot compensate for loss of NFAT5 recruitment to the viral LTR in the context of MTb co-infection.

Given that the NF-κB and NFAT5 binding motifs overlap, how do NF-κB and NFAT5 function at this overlapping or shared site in the LTR to drive viral transcription in response to MTb infection? Because NFAT5 expression continues to rise for at least 48 hours post-MTb infection (Figure 2A), one possibility is that the host factors NF-κB and NFAT5 associate with the LTR at different stages after viral activation. While NF-κB presence in the nucleus declines in the hours post MTb infection [74], [75], NFAT5 levels escalate. Thus, NFAT5, which is pivotal for HIV-1 transcription in unstimulated cells [31], may, after Mtb infection, perpetuate viral transcriptional initiation begun by NF-κB and, with Tat levels also elevated, promote high levels of sustained HIV-1 transcription. Indeed, in response to hypertonic stress, NFAT5 binds to the aldose reductase (AR) promoter and induces rapid hyperacetylation of histones H3 and H4 [76]. Furthermore, hypertonic stress of mouse renal collecting duct epithelial cells results in NFAT5 and NF-κB complex formation at NF-κB-dependent gene promoters [77]. Thus, there are precedents for imagining that NFAT5 could function at the HIV-1 LTR by inducing histone remodeling in a manner similar to NF-κB ([69], reviewed in [68], [70]) or by directly interacting with NF-κB in a cooperative manner.

In conclusion, we have demonstrated that NFAT5 is required for the replication of R5-tropic subtype B and subtype C HIV-1 isolates in response to MTb-co-infection of human PBMC and MDM. Functional NF-κB interaction with the viral LTR is also required, but fully intact NF-κB binding elements are unable to compensate for the loss of NFAT5 recruitment to the viral promoter. In addition, we have demonstrated that MTb infection or stimulation with the TLR2 ligand PAM3Cys, induces NFAT5 gene expression in human monocytes. Furthermore, we have shown that MTb stimulation of NFAT5 depends on TLR pathway signaling molecules, including MyD88, IRAK1, and TRAF6. Taken together, the findings presented here enhance the general understanding of the innate immune response to MTb infection by showing that NFAT5 is a major mediator of TLR-dependent gene expression; its importance for gene regulation is likely applicable to other MTb- and TLR-regulated genes. Moreover, these data provide molecular insights into MTb regulation of HIV-1 transcription, thereby elucidating several new targets for therapeutic interventions aimed at controlling TB/HIV-1 co-infection.
